# Genetic Characterization of Rubella Virus Strains Detected in Spain, 1998-2014

**DOI:** 10.1371/journal.pone.0162403

**Published:** 2016-09-13

**Authors:** Alex O. Martínez-Torres, María M. Mosquera, Fernando De Ory, Alejandro González-Praetorius, Juan E. Echevarría

**Affiliations:** 1 Centro Nacional de Microbiología, Instituto de Salud Carlos III, Majadahonda, Madrid, Spain; 2 Laboratorio de Microbiología Experimental y Aplicada, Vicerrectoría de Investigación y Post-Grado, Universidad de Panamá, Ciudad de Panama, Panama; 3 Sección de Microbiología, Hospital Universitario de Guadalajara, Guadalajara, Spain; 4 CIBER en Epidemiología y Salud Pública, CIBERESP, Madrid, Spain; Oklahoma State University, UNITED STATES

## Abstract

The National Plan for the Elimination of Rubella was implemented in Spain in 2008 using the logistics of the National Plan for the Elimination of Measles that have been employed since year 2000. Molecular characterization of rubella virus (RUBV) is important for disease surveillance and for monitoring elimination of the disease throughout the world. We describe the first complete series of data regarding the circulation of RUBV genotypes in Spain. The 739-nucleotide fragment designated by the WHO for RUBV genotyping was sequenced in 88 selected cases collected from 1998 to 2014. Five genotypes were identified: 1E, 2B, 1J, 1I, and 1a. Genotype 1E was predominant between 1998 and 2003 but was replaced by genotype 2B, which was detected in sporadic cases in 2004, 2006, 2008, 2012, 2013 and 2014. There was an outbreak of genotype 2B in Algeciras (Andalusia) in 2008. Genotype 1J caused an outbreak in Madrid in 2004/2005 and sporadic cases in 2005 and 2007. Genotype 1I was found to have infected an immune-suppressed patient with neurological symptoms in 2008. Finally, vaccine strain RA 27/3 was detected in three sporadic cases, two of them immune-suppressed and without a recent history of vaccination. This suggests that during these years there were a series of imported sporadic cases and outbreaks, confirming the findings of epidemiological data analysis. The importation sources were generally consistent with our geographic and cultural ties, mainly with Europe (genotypes 1E, 2B, 1I) and Latin America (1J).

## Introduction

Rubella virus (RUBV) usually causes a mild exanthematic disease, frequently accompanied by adenopathy, and occasionally by arthralgia. Complications of this infection are rare and include encephalopathy and thrombocytopenia. However, the most severe consequence of this virus is the congenital rubella syndrome (CRS) when the infection occurs in pregnant women, particularly during the first trimester of pregnancy [1].

RUBV is a serologically monotypic virus. However, it displays enough genetic variability to distinguish two clades, designated A and B, which include 12 recognized genotypes (1B, 1C, 1D, 1E, 1F, 1G, 1H, 1I, 1J, 2A, 2B, 2C) and a single provisional one (1a). This classification was initially proposed in 2005 [2], and revised in 2007 [3] and 2013 [4], and relies on the sequencing of a fragment of 739 nucleotides (nt 8731–9469) from the gene coding for the envelope protein E1. This sequence encodes amino acids (aa) 159–404 (of 481) of the E1 protein. Although our knowledge of the geographic distribution of rubella genotypes has grown substantially, those present in many countries and regions remain unknown [5], even though rubella is still recognized as a globally important disease in the general public health context [6]. This knowledge is crucial for tracing the source of imported outbreaks in countries that are close to eliminating rubella. Moreover, the pattern of circulation of RUBV genotypes is by itself an indicator of the stage a territory has reached on the way to rubella elimination.

In Spain, monovalent rubella vaccine was introduced in the late 1970s, when it was administered to 11-year-old girls in schools [7]. In 1981, one dose of the measles/mumps/rubella (MMR) combined vaccine was introduced in the regular immunization schedule at the age of 15 months for all children. In 1996, a second dose at 11 years was introduced [8]. After 1999, this second dose was given to four-year-old children [9]. At the national level in Spain, vaccine coverage for rubella exceeds 95.3% for the first dose and 90.7% for the second dose [10]. As a result of this vaccination program, the annual rate of incidence dropped by several orders of magnitude, from hundreds in 1982 to fewer than one case per 100,000 habitants in the first years of this century. Nevertheless, the pattern is very different in other regions of the world and rubella infection remains endemic in many areas, or has been only recently targeted for elimination, as is the case in Latin America [11]. The rubella vaccine was only introduced in Latin American countries in the late 1990s, so many adult immigrants from those countries who are resident in Spain are not immunized. These circumstances led to a small outbreak in Madrid in 2003 [12], a larger one in 2004–2005 [13, 14, 15], and another one in Catalonia in 2005 [16] among Latin American immigrants and Spanish males born before the universalization of the vaccine. The characterization of the genotype 1J strain causing the outbreak in Madrid in 2005 [13] remains the only historical data of RUBV genotype circulation in Spain.

In this report, we describe the RUBV genotypes detected in Spain over a period of 17 years, between 1998 and 2014, with the aim of establishing the pattern of virus circulation and testing the hypothesis of the interruption of endemic circulation suggested by the incidence data. This represents the first series of data obtained at national level.

## Materials and Methods

### Clinical samples

Eighty-eight clinical samples (nine of urine, eight of serum, three of blood, two of saliva, two of cerebrospinal fluid [CSF], one isolate and 63 pharyngeal exudates) from 88 confirmed rubella cases collected from 1998 to 2014 in several regions of Spain were studied (Tables 1 and 2).

**Table 1:** RUBV Genotypes found in Spain, 1998/2014.

Region	1998	2003	2004	2005	2006	2007	2008	2009	2012	2013	2014
**Andalusia**			2B ^b^	1J ^b^			2B ^a^	1E ^b^			2B ^b^
**Aragón**									2B ^b^	1a ^b^	
**Canary Islands**	1E ^b^										
**Castile-La Mancha**							1I ^b^			2B ^b^	
**Catalonia**						1J ^b^					
**Ceuta**							2B ^b^				
**Madrid**		1a ^b^, 1E ^a^	1J ^a^	1J ^a^	2B ^b^		2B ^b^	1a ^b^	2B ^b^		
**Valencia**							2B ^b^		2B ^b^		

^*a*^ Genotypes in outbreaks.

^*b*^ Genotypes in sporadic cases.

**Table 2:** GenBank accession numbers of RUBV sequences found in Spain, 1998/2014.

Sequences	GenBank accession numbers	Year	City	Genotype
**0947570_1**	KU601196	2009	Madrid	1a
**1085F03**	KU601203	2003	Madrid	1E
**69F04**	KU601197	2004	El Ejido (Almería)	2B
**165F03**	KU601198	2003	Madrid	1a
**16815Zaragoza2012 (4)**	KU601215	2012	Alcañiz (Teruel)	2B
**24433Madrid2012**	KU601216	2012	Madrid	2B
**2585Castile-LaMancha2013**	KU601210	2013	Guadalajara	2B
**11520Granada2014 (2)**	KU601213	2014	Granada	2B
**16720Madrid2012**	KU601214	2012	Madrid	2B
**941536**	KU601219	2009	Madrid	1a
**1897F05**	KU601208	2005	Seville	1J
**941531**	KU601218	2009	Madrid	1a
**1329F08**	KU601206	2008	Madrid	2B
**0928382F**	KU601217	2009	Malaga	1E
**1902F08**	KU601209	2008	Alicante	2B
**3819F06**	KU601211	2006	Madrid	2B
**921I08**	KU601201	2008	Guadalajara	1I
**1151I08A**	KU601205	2008	Guadalajara	1I
**1151I08**	KU601204	2008	Guadalajara	1I
**1854F07**	KU601207	2007	Barcelona	1J
**216C98**	KU601199	1998	La Laguna (Tenerife)	1E
**5495F08**	KU601212	2008	Madrid	2B
**776F08 (17)**	KU601200	2008	Algeciras (Cádiz)	2B
**1083F03**	KU601202	2003	Madrid	1E
**277E (30)**	EU518607	2005	Madrid	1J
**577E**	EU518608	2005	Madrid	1J
**581E**	EU518609	2005	Madrid	1J
**701E**	EU518610	2005	Madrid	1J
**719E**	EU518611	2005	Madrid	1J
**825E**	EU518612	2005	Madrid	1J
**837E**	EU518613	2005	Madrid	1J
**856E**	EU518614	2005	Madrid	1J
**888E**	EU518615	2005	Madrid	1J
**896E**	EU518616	2005	Madrid	1J
**1247E**	EU518606	2005	Madrid	1J

The numbers in parentheses represent the number of sequences.

### Ethics statement

Specimens were collected at origin and processed in the National Center of Microbiology in accordance with WHO recommendations [17]. All samples were anonymized.

The project was approved during its evaluation by the Technical Commission of Evaluation of the Instituto de Salud Carlos III (ISCIII) and the National Agency of Evaluation and Prospective.

### Viral isolation in cell culture

Isolation was performed as previously described [13, 18] in Vero and Vero E6 cells (from the ISCIII collection).

### RT and amplification

Total nucleic acids were extracted from samples and a fragment of 875 bp including the region of E1 gene, recommended by the WHO for RUBV sequencing, was amplified as previously described [13]. Sequences were aligned and analyzed together with WHO reference strains [3, 4] to determine the genotype. Finally, phylogenetic trees were obtained for each detected genotype including other RUBV sequences obtained from GenBank. Sequence management and phylogenetic constructions were carried out as previously described [13, 19].

## Results

### Genotype assignation of sequences obtained from samples

**Fig 1:**
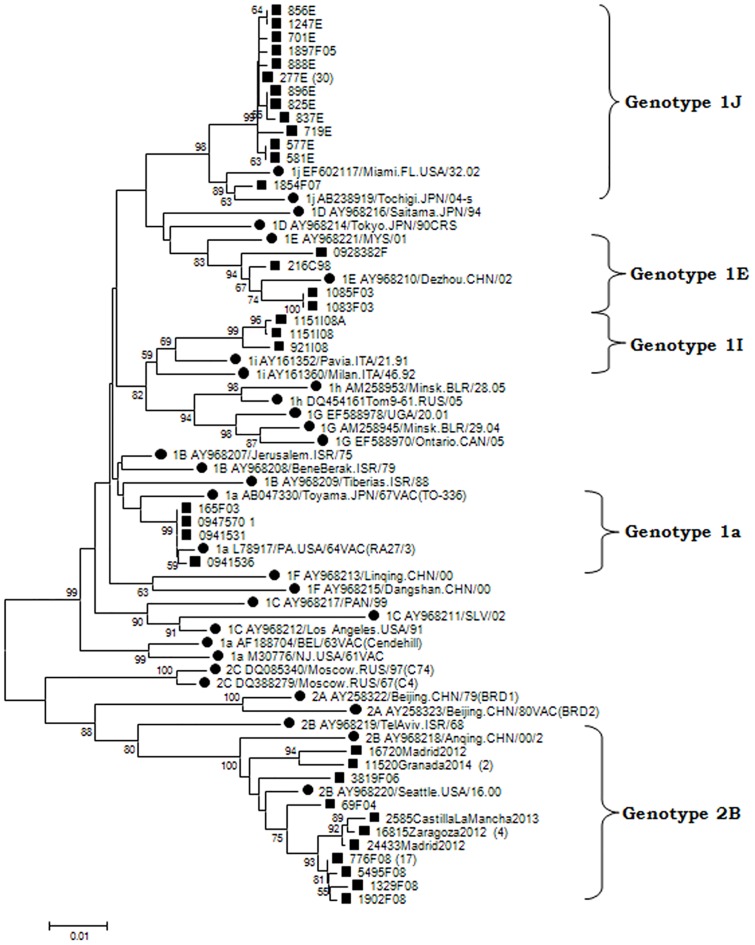
Phylogenetic relationships among RUBV genotypes. The phylogenetic tree was obtained by Neighbor-Joining analysis. (Black circle), reference strains; (Black square), strains detected during this study. The numbers in parentheses represent the number of sequences.

**Fig 2:**
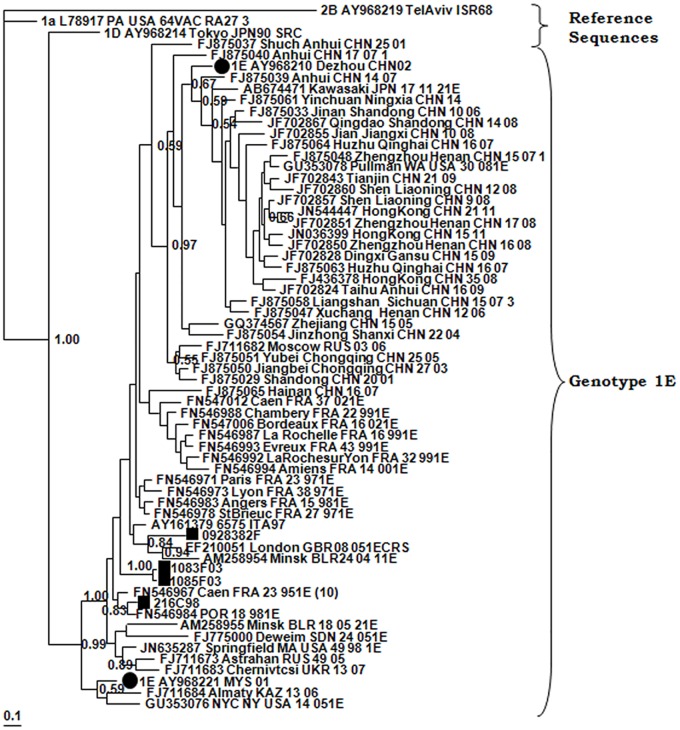
Phylogenetic relationships among genotype 1E strains. The phylogenetic tree was obtained by Bayesian inference. (Black circle), reference strains; (Black square), strains detected during this study.

Five genotypes were detected (1E, 2B, 1J, 1I and 1a) in sporadic cases or outbreaks (Table 1; Fig 1). Genotype 1E was detected in 1998 (Canary Islands) and in 2003 was associated with an outbreak in Madrid [12]. It was the only genotype known to be circulating in Spain during this period. It did not produce another sporadic case until 2009, in Malaga (Andalusia) (Table 1). When analyzed with other 1E sequences available in GenBank (Fig 2), all the sequences were found to be located at the base of the phylogenetic tree along with others from France, Italy, United Kingdom, Belarus and Portugal from the same years. In particular, sample 216C98 from the Canary Islands was grouped with a sequence detected the same year in Portugal, and sample 200928382 was grouped with sequences from the UK and Belarus. These results suggest that all the imported strains of genotype 1E probably originated in Europe.

The GenBank accession numbers of RUBV sequences found in Spain, 1998/2014, are shown in Table 2.

**Fig 3:**
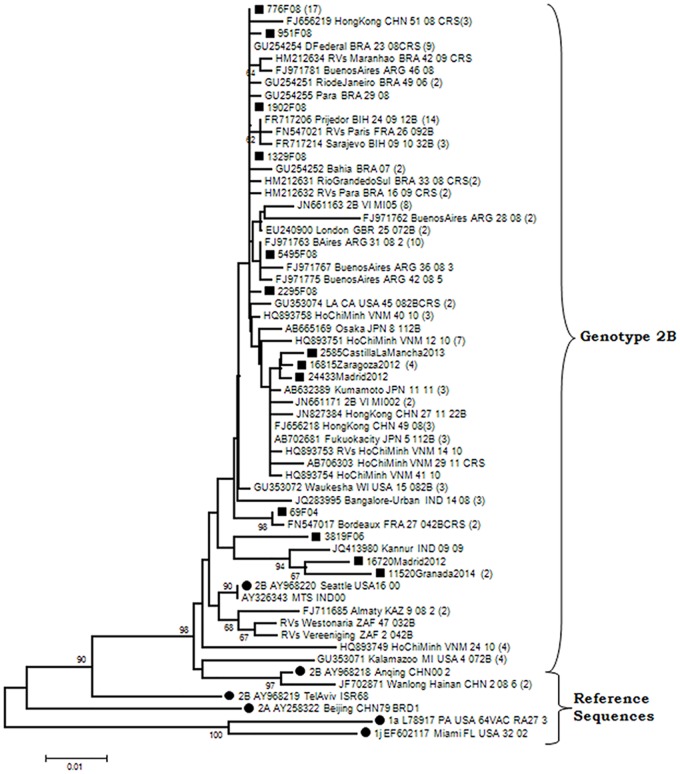
Phylogenetic relationships among genotype 2B strains. The phylogenetic tree was obtained by Neighbor-Joining analysis. (Black circle), reference strains; (Black square), strains detected during this study.

Only sporadic detections of genotype 2B in El Ejido (Almería, Andalusia) in 2004 and Madrid in 2006 had been described until the appearance of an outbreak in Algeciras (Cádiz, Andalusia) in 2008, with associated cases arising in Madrid, Ceuta (a Spanish city located on the coast of North Africa) and Valencia. Other sporadic cases were detected in Zaragoza, Madrid and Valencia in 2012, Castile-La Mancha in 2013, and Granada (Andalusia) in 2014 (Table 1; Fig 2). All the Spanish sequences of genotype 2B, including those from the outbreak in Algeciras (2008), represented by sequences 776F08 (17 equal sequences) and 951F08, occupied a basal position in the phylogenetic tree with others found in GenBank collected from several very different locations. Consequently, as it seems to be widespread around the world, it is difficult to establish the geographical origin of the imports with certainty. Only sequence 69F04 showed any significant association with sequences detected the same year in France (FN547017/Bordeaux.FRA/27.04SRC). Also, two sequences from Granada 2014 and Madrid 2012 grouped with an Indian sequence (JQ413980 RVI/Kannur-IND/09-09) with a significant bootstrap value of 94 (Fig 3). Consequently, strains of genotype 2B that seemed to be circulating worldwide were frequently imported.

**Fig 4:**
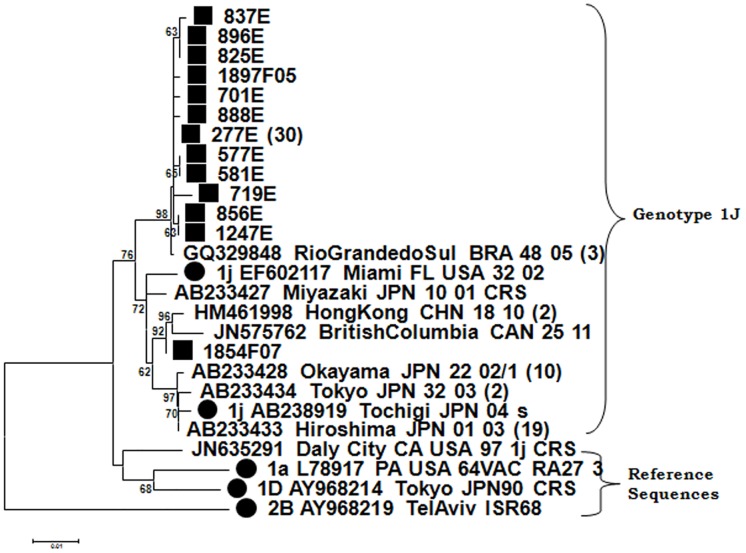
Phylogenetic relationships among genotype 1J strains. The phylogenetic tree was obtained by Neighbor-Joining analysis. (Black circle), reference strains; (Black square), strains detected during this study.

A strain of genotype 1J caused a large outbreak in Madrid of about 460 cases in 2004–2005, mainly affecting the Latin-American community [13, 14, 15], and a sporadic case was recorded in Seville (Andalusia). The associated sequences seem to be related to three others detected in Brazil in the same year (CQ329848/RioGrandedoSul.BRA/48.05) (Fig 4). One of them even showed a complete identity, suggesting importation from South America as the most probable origin of the outbreak, given the origin of most of the affected population. Sequence 854F07, detected two years later in Barcelona (Catalonia) was grouped in a different branch with two sequences, one from Hong Kong (HM461998/Hong Kong.CHN/18.10) in 2010 and the other from Canada (JN575762/BritishColumbia.can/25.11) in 2011, suggesting a different importation event from a different source.

**Fig 5:**
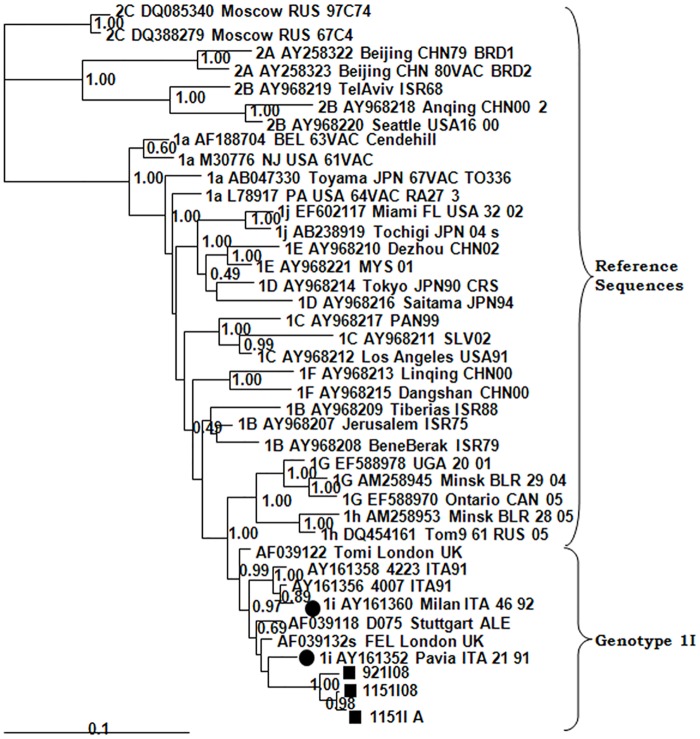
Phylogenetic relationships among genotype 1I strains. The phylogenetic tree was obtained by Bayesian inference. (Black circle), reference strains; (Black square), strains detected during this study.

A sporadic case produced by genotype 1I was detected in Guadalajara (Castile-La Mancha) in 2008. The homology observed among the sequences associated with this case and reference strains ranged between 96.2% and 97.4% for 1I, and from 88.9% (2A) to 96.4% (1B) across other genotypes (data not shown). The sequences analyzed were grouped with 1I reference sequences with a significant posterior probability of 0.97 in the Bayesian analysis (Fig 5). The patient was immunocompromised, since she had the underlying disease of recessive autosomal polyclonal agammaglobulinemia. In March 2008 she was negative as shown by RT-PCR, for measles, rubella and parvovirus B19 [18], and had high avidity-specific RUBV IgG, which excluded a primary recent rubella infection. On May 14, RT-PCR gave positive results for RUBV RNA from serum and cerebrospinal fluid (CSF) coincident with neurological symptoms. Two serum samples and a pharyngeal swab taken later on July were also positive for RT-PCR. It proved possible to isolate RUBV from a cell culture of this last sample. The sequences obtained from the various samples of this patient differed at as many as eight positions (Table 3). The possible source of infection is unknown because no sequences of this genotype were available for that time in GenBank. The presence of genotype 1I is known in Europe (Italy, Germany, and England) from the late 1980s and the beginning of the 1990s, and it was detected for the last time in Italy in 1994. It has been considered inactive since then [10].

**Table 3:** Differences in nucleotide and amino acid sequences obtained directly from pharyngeal exudates and spinal fluid or isolated from pharyngeal exudate of a patient with genotype 1I. Position refers to the sequenced fragment.

Sequences	Dates	Clinical samples	Nucleotide Position									
			37	55	190	244	247	430	487	523	562	721
**921I08**	05/22/2008	CSF	T	C	A	C	C	C	T	G	T	G
**1151I08**	07/3/2008	Phar Ex	C	T	A	T	T	T	C	G	C	A
**1151I08**	07/15/2008	Isolate	C	T	G	T	T	T	C	C	C	A

**Fig 6:**
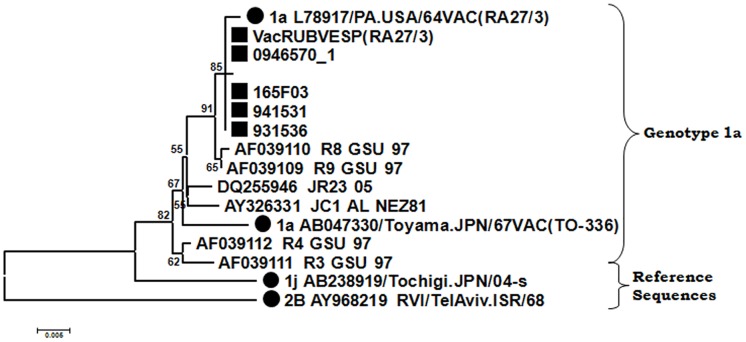
Phylogenetic relationships among genotype 1a strains. The phylogenetic tree was obtained by Neighbor-Joining analysis. (Black circle), reference strains; (Black square), strains detected during this study.

Finally, three cases were detected in Madrid in 2009 associated with strains with sequences identical to the RA 27/3 vaccine strain used in Spain (Fig 6). A highlight was that two of these three cases were immunocompromised individuals (no such information was available about the third case). There is no evidence of recent vaccination against rubella in any of these cases.

## Discussion

Genotyping is included in the WHO protocols for epidemiological surveillance of rubella as a relevant indicator of the stage of elimination [2, 3, 4, 20] and a tool for tracing chains of transmission, identifying sources of infection [13, 20] and confirming vaccine-associated cases [21]. This work reports the first complete series of data of RUBV genotype circulation in Spain, covering a 17-year period, and adding information to previous findings [13]. Due to the low incidence of rubella during the years of the study the number of cases is not high. We were unable to amplify viral RNA from a large collection of positive rubella IgM sera from the last 30 years, stored at -20°C and frozen and thawed an unknown number of times for serological studies (data not shown). During the early years (1998–2003) genotype 1E was most frequently detected, probably having been imported from other European countries that also experienced concomitant cases caused by this genotype [22, 23]. Genotype 2B subsequently replaced genotype 1E as the most frequently detected genotype. However, a large outbreak in 2005 was caused by genotype 1J. These occurrences suggest a pattern of sporadic imports without establishment of endemic circulation by any genotype (Table 1; Fig 1). The pattern is consistent with the extremely low incidence of rubella detected during these years and the many periods free of cases longer than the incubation time of the disease [24]. All this suggests a lack of indigenous circulation and a situation approaching the goal of elimination. However, as the existence of local clusters of susceptible adults cannot be discounted, imported cases and even self-limiting outbreaks could consequently occur as long as endemic circulation continues somewhere in the world. The origins of imported rubella cases seem to have been different. For the most frequently detected 1E and 2B genotypes, Europe seems to be the most frequent origin, although it is usually difficult to establish. The genotype 1J outbreak, which occurred in Madrid in 2004–2005 [13, 14, 15], highlights the importance of generating sequence data about rubella genotypes for epidemiological purposes. It affected about 460 patients, predominantly Latin American immigrants, and adult Spanish males born in the early 1980s (around the time the MMR vaccine was introduced [15]), who had not been immunized in previous campaigns that had focused solely on 11-year-old girls [13, 14, 15]. In the original description of the Madrid outbreak [1, 22] there was no evidence of the presence of genotype 1J in either Europe or South America, and the origin of the outbreak could not be traced. After these papers were published, several Brazilian 1J sequences from the same year, some of them identical to those detected in the Madrid outbreak, were published. These new data indicated that the most probable source of this outbreak was South America, the original provenance of most of the patients. Although there was no evidence of recent travel to South America for any of the cases detected earlier, an index case with a recent travel history could have been missed, since mild or subclinical rubella is frequent [21, 25]. This highlights the importance of molecular epidemiology on tracing the origin of rubella outbreaks and the necessity of generating enough sequencing information available in databases such as RubeNs. Another 1J strain detected two years later in a sporadic case from Catalonia is more similar to strains 1J of Asian origin, but different from those responsible for the outbreak in Madrid.

The last rubella outbreak included in this study occurred in Algeciras (Cádiz) in 2008 and was produced by genotype 2B. The origin is unknown and difficult to establish on the basis of sequencing data, since there is evidence of circulation of this genotype in many parts of the world at the same time. Phylogenetic trees show the strains of this outbreak in a branch including sequences from very different origins as China, Vietnam, Japan, Argentina, Brazil, USA, Bosnia-Herzegovina, England and France. The origin of the outbreak was attributed to a cruise connecting the Spanish city of Algeciras with Morocco, although there was no information available about rubella genotypes in Morocco at that time. It is of particular note that these clinical rubella cases were initially missed because they were detected in the context of a concomitant measles outbreak. They could be discriminated only on the basis of laboratory diagnosis [26]. This illustrates the importance of the laboratory in the epidemiological surveillance of vaccine-preventable viral rashes and, in particular, the necessity either of double measles/rubella testing of initially negative cases or the use of multiplex genomic amplification techniques, as was the case in this event.

The most surprising finding about the presence of rubella genotypes in Spain described in this study is the case caused by genotype 1I, since this was considered to be inactive throughout the world [20]. This genotype circulated in Europe (Germany, England, and Italy) between 1986 and 1994, but has not been reported since then [1, 6]. The virus was isolated in a 24-year-old immune-compromised patient. The sequences were obtained from different locations as CSF, serum and throat swab (Fig 5) and showed as many as eight differences among them (Table 3). This is almost the same number of variations as were generated over a year during the outbreak of genotype 1J in Madrid, which affected hundreds of people. It suggests the existence of a heterogeneous population within the patient that should have taken a long time to be generated in the course of a persistent infection established at any time in the past. The age of the patient is compatible with a primary infection during the time when the virus was circulating in other European countries such as Italy, France and Germany. The persistence of the infection in the patient could be explained by their immune status. During this study the RA27/3 vaccine strain was detected in four cases, none of which had a recent history of vaccination. The sequence is the same as that obtained from vaccine marketed in Spain. In a previous study, RUBV could be detected in three of 26 throat swab samples from children taken 10 to 14 days after vaccination. One of them was even recovered in culture [27]. This study showed evidence of asymptomatic excretion of RA 27/3 vaccine strain after vaccination, while the four cases analyzed in our study also suggest the possibility of secondary transmission. Moreover, it is noteworthy that two of these patients were immunosuppressed and the virus could be detected in as many as eight samples of one of them (data not shown). Unlike the case described above, which was caused by genotype 1I, the sequences showed only a single variation, which is compatible with a recent infection.

In conclusion, we detected five different genotypes of RUBV (1E, 2B, 1J, 1I and 1a) in Spain between 1998 and 2014, although there was no evidence of the continuous circulation of any of them. This is consistent with an advanced stage on the way towards rubella elimination in Spain, as suggested by the extremely low incidence. Europe and Latin America seem to be the most common sources of importation of rubella in Spain, in accordance with the very close geographical, social and cultural ties with the country. The discovery that a genotype considered extinct until then had caused persistent infection called into question the role of the immune-compromised as a source of RUBV after elimination had been achieved. Finally, the possibility of secondary transmission of vaccine virus among immune-compromised patients also requires further study. Further global efforts to genotype RUBV are necessary to obtain a more accurate picture of the global distribution of RUBV genotypes and the molecular epidemiology of RUBV. It would also allow a better management of outbreaks and a more accurate evaluation of worldwide progress towards the elimination of rubella.
